# Highlight: Epigenetics Help *Hydra* Get Ahead

**DOI:** 10.1093/gbe/evab250

**Published:** 2021-12-08

**Authors:** Casey McGrath

The Hydra of Greek and Roman mythology was a multiheaded serpent with the peculiar ability to regrow its heads when they were cutoff. Named after this mythological creature, the genus *Hydra* consists of small, freshwater animals in the phylum Cnidaria that are related to jellyfish, sea anemones, and corals. Although these 10-mm long animals seemingly have little in common with their namesake, they do share one important feature: cutoff the head of a *Hydra*, and it will grow a new one. In a new study titled “Coordinated gene expression and chromatin regulation during *Hydra* head regeneration” published in *Genome Biology and Evolution*, researchers at the University of California Irvine show that head regeneration is regulated in part by epigenetics, a term that refers to reversible changes that do not affect the DNA sequence but influence the way genes are expressed (Murad et al. 2021). The study finds that head regeneration involves developmentally programmed remodeling of the DNA around regulatory elements, pointing to an ancient origin of this type of regulation. 

**Fig 1.**
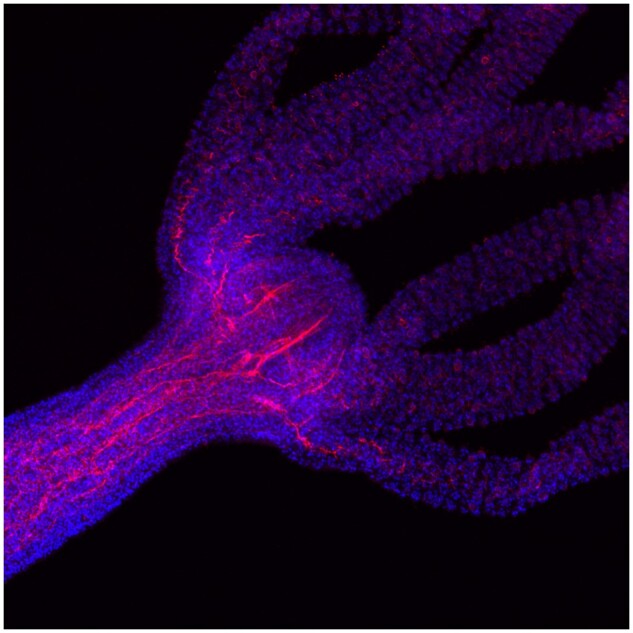
*Hydra* hypostome and tentacles. The hydra head consists of a hypostome (area surrounding the mouth) and tentacles. This confocal image shows DAPI (blue), which stains nuclei, and an antibody raised against acetylated alpha tubulin (red), which stains neurons. Image courtesy of Dr David Plachetzki.

An adult hydra has a simple body plan with a head at the top and a foot at the bottom, joined by a central tube. In the middle are stem cells that continue to divide throughout the hydra’s life, constantly displacing existing cells toward each end, where dead cells are sloughed off. A group of 30–500 cells at the tip of the head, collectively referred to as the “head organizer,” is responsible for signaling the incoming stem cells to differentiate into head structures, including a mouth and the poison-containing tentacles the hydra uses to stun prey and capture food (fig. 1). After decapitation, the head organizer reforms first and subsequently directs development of the remaining head structures.

According to the study’s cofirst author Aide Macias-Muñoz, the purpose of the study was to understand the evolution of the systems that regulate developmental programs and to compare those present in cnidarians with those observed in bilaterians, a group of animals with bilateral symmetry which includes humans, earthworms, fruit flies, and sea stars. “We know a lot about the genes and regulatory networks that underlie development in model bilaterians, but not much is known in other organisms. Cnidaria is a unique group to investigate complex traits as it is the sister to Bilateria. Thus, we can learn about the genes and pathways that were present before the Cnidaria/Bilateria split.”

Cofirst authors Rabi Murad and Macias-Muñoz, along with senior author Ali Mortazavi, hypothesized that the regulation of head regeneration may be similar to that of budding, a form of asexual reproduction in *Hydra* in which a head organizer is formed in the early stages. The similarities between the initial stages of head regeneration and budding led the authors to expect similar patterns of gene expression between budding and regenerating tissue. To test this, the authors isolated cells from both budding and regenerating hydra at different time points and sequenced the transcriptomes of the resulting samples to get a genome-wide look at gene expression patterns during these two processes.

**Fig. 2.**
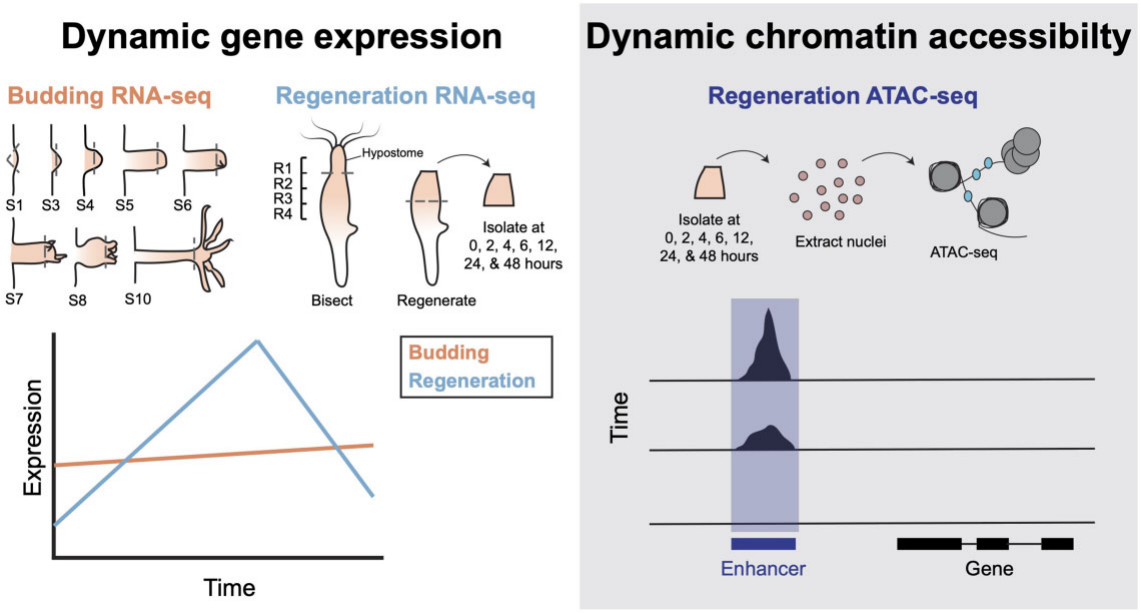
Complex enhancer activity in *Hydra* during regeneration. *Hydra* have dynamic gene expression during regeneration, with less dynamic changes during budding. Areas of open chromatin are also dynamic during a time course of regeneration.

Surprisingly, the study’s results showed quite distinct patterns across these two developmental programs (fig. 2). According to Macias-Muñoz, “One of the interesting findings in our study is that changes in gene expression are different between budding and regeneration. We actually find that many genes steadily increase in expression during budding, while expression during regeneration is very dynamic, with significant shifts in high and low expression.”

To further investigate the regulatory landscape of head regeneration, the authors assessed the presence of two epigenetic marks—histone modifications and open chromatin—across the *Hydra* genome. Histone proteins act as the scaffolding around which DNA is folded in order to compact it into a form known as chromatin. Modifications to histone proteins enable the chromatin to unwind at specific locations in the genome, making certain DNA sequences accessible to transcription factors that regulate gene expression. By evaluating histone modifications and regions of open chromatin, the authors identified regions of the genome that are likely to be involved in the regulation of this developmental event. Based on their data, the authors predicted nearly 10,000 candidate promoters and 3,000 candidate enhancers that potentially regulate gene expression during head regeneration.

The open chromatin and histone modification maps suggested that extensive and dynamic remodeling of the genome drives changes in gene expression associated with head regeneration in *Hydra*. Macias-Muñoz notes, “It is not surprising that changes in chromatin structure accompany and likely drive differences in gene expression. What is exciting is that we find this level of complexity in a member of the Cnidaria.” This demonstrates that complex epigenetic regulatory mechanisms predate the split of cnidarians from bilaterians, suggesting that this form of regulation is even more ancient than previously believed. Adding to this evidence, Macias-Muñoz notes that there is overlap between the two lineages in some of the specific enhancer motifs used: “The areas that have dynamic open chromatin in *Hydra* are enriched for transcription factor motifs that are proposed to have conserved roles in bilaterian development.”

The study’s authors point out that additional studies are needed to confirm these findings. “One limitation of the current study,” notes Macias-Muñoz, “is that we have not yet performed functional validation for many of the genes and transcription factors that we find to have dynamic expression and binding.” Although the authors hope to validate the coexpression and function of candidate genes and transcription factors underlying regeneration in the future, this is likely to be a significant undertaking. “A foreseeable obstacle in validating gene function is that many of these genes are essential, so timing and localization of genetic perturbation will have to be carefully considered,” says Macias-Muñoz.

Another avenue for future exploration is to investigate the regulatory mechanisms underlying other types of regeneration in both *Hydra* and in other cnidarians. In addition to regenerating their heads, hydra can regenerate their feet and can even form polyps from cellular aggregates derived from disassociated tissues. Moreover, Macias-Muñoz notes that “Some jellyfish can regenerate their eye-bearing tissues called rhopalia. I would like to investigate what cellular and molecular mechanisms are underlying that regeneration as well.” 
